# Note on the Delivery of the Placenta

**Published:** 1882-11-20

**Authors:** Charles Jewett


### NOTE ON DELIVERY OF THE PLACENTA.

By CHAS. JEWETT, M. D.

The diversity of usage which still obtains in the management of the third stage of natural labor prompts this brief note, in the hope of eliciting the views and practice of the Society.

The points to which I wish especially to invite attention are the method and the time of placental delivery. These comprise a larger part of the treatment of the placental stage, the management of which is, perhaps, the most important office of the obstetrician in the conduct of natural labor.

The preferred method of delivering the placenta is that of Crede, of Leipsig. In Germany this method is now used to the exclusion of almost every other, and it would seem that so eminently judicious and rational a procedure could not fail of universal adoption. That such is not yet the case is possibly due to the fact that in many of the standard works on obstetrics the Credean method, if mentioned at all, is more or less imperfectly stated. The Credean procedure is not identical, as claimed by Barnes, with that taught by Hardy and McClintock, and long practiced in England. It differs essentially from the practice of the Dublin school, which consists in expelling the afterbirth by crowding the uterus downward in the pelvic cavity by pressure upon the fundus. It does not contemplate the mere expression of the placenta by compressing a uterus still flaccid from inertia, as might be inferred from the statement of one of the latest text-books. On the contrary, the essence of the German method is compression of the uterus during contraction, and with but slight downward pressure.

The Credean method is practiced as follows: The obstetrician, laying his hand flat upon the abdomen of the patient, stimulates the uterus to contract by moving the abdominal wall in a circular manner over the uterus. The friction, gentle at first, is increased till the uterus contracts. At the height of the uterine contraction the upper segment of the uterus is firmly grasped with the hand, the fingers over the posterior, and thumb over the anterior surface. The placenta is thus expressed from the uterus, only enough downward pressure being used in the axis of the uterus to maintain a firm grasp. Failing in the first attempt, the compression may be repeated with each uterine contraction till successful, friction being continued meantime to maintain the retraction thus far accomplished, and to provoke further uterine efforts.

It is the peculiar merit of this method that it, more closely than any other, imitates the natural process of placental expulsion. Moreover, it is designed to supplement the expulsive efforts of the uterus, not to replace them. It maintains firm retraction of the uterus till the afterbirth is expelled, keeps the uterine vessels securely ligated, and prevents the formation of deep coagula in the uterine sinuses. It favors, more than any other plan, permanent uterine retraction after the delivery of the placenta. Potent for good, it is incapable of harm.

Some practice is undoubtedly necessary to the utmost facility in this procedure, but the knack once acquired, other measures will be very rarely called for.

With reference to the time of placental delivery, the prevailing practice, in the judgment of the writer, favors too long delay. Dr. Playfair, following the teachings of McClintock, says that no attempt should be made at delivery of the placenta till twenty minutes after the expulsion of the child. Certain other obstetric writers sanction even longer delay. The arguments of Dr. Playfair in support of his practice are that time is thus allowed for recovery from the shock or exhaustion of the second stage, for the separation of the placenta, and for the formation of coagula in the uterine sinuses. While these would be valid reasons for delay under the old practice of placental extraction, they do not forbid early resort to the Credean method. As a rule, after the ligation of the cord, the sooner the uterus can be made to cast out the placenta the better for the patient.

There is surely no exhaustion of the uterus when it can be provoked to contract by gentle friction. Again, the very agency by which separation of the placenta is accomplished is uterine contraction and retraction. Against the dangers of post-partum hemorrhage the chief security lies in the ligation of the uterine vessels by retraction of the muscular structures. Coagula in the uterine sinuses are a feeble barrier against hemorrhage. Moreover, thrombi extending into the intermuscular portion of the uterine veins are a positive source of danger, from their liability to infection. Promptness, again, facilitates delivery. By too long waiting the way may be narrowed by the contraction of Bandl's ring, and the difficulty of expulsion be thus increased. As a rule, then, the placenta should be expelled as soon as its function is ended; that is, as soon as the infantile circulation is established and the cord divided.

A word with reference to the use of ergot may not be out of place. Prof. Lusk disparages the exhibition of this drug before the afterbirth is delivered, owing to its tendency to induce so-called hour-glass contraction. In my practice a drachm of the fluid extract ot ergot is given by the mouth in every case—or its equivalent hypodermically—as the head passes the vulva. Under the above management of the placenta, it is expelled before the effect of the drug is develeped. I should be unwilling to sacrifice the advantage gained by prompt and complete retraction of the uterus, through fear of a possible danger so seldom realized.

In conclusion I submit the following summary:

Use constant friction or uterine massage after the delivery of the head, for the double purpose of maintaining retraction and provoking uterine effort.

Supplement the uterine efforts, if need be, by compression.

After the placental expulsion, continue friction till retraction is complete and permanent.

Use ergot on the birth of the head to promote the prompt and perfect completion of the third stage.

Aim to deliver the placenta, as a rule, directly after, not before, the ligation and division of the cord.—*Society County of Kings.*

